# Prescribing style and variation in antibiotic prescriptions for sore throat: cross-sectional study across six countries

**DOI:** 10.1186/s12875-015-0224-y

**Published:** 2015-01-29

**Authors:** Gloria Cordoba, Volkert Siersma, Beatriz Lopez-Valcarcel, Lars Bjerrum, Carl Llor, Rune Aabenhus, Marjukka Makela

**Affiliations:** The Research Unit for General Practice and Section of General Practice, Department of Public Health, University of Copenhagen, ØsterFarimagsgade 5, P. O. Box 2099, DK-1440 Copenhagen, Denmark; Universityof Las Palmas de Gran Canaria, Campus Universitario de Tafira, Las Palmas de GC, CanaryIslands, Spain; University Rovira i Virgili, Spanish Society of Family Medicine, Primary Healthcare Centre Jaume I, Tarragona, Spain; Finnish Office for Health Technology Assessment, National Institute for Health and Welfare, Helsinki, Finland

## Abstract

**Background:**

Variation in prescription of antibiotics in primary care can indicate poor clinical practice that contributes to the increase of resistant strains. General Practitioners (GPs), as a professional group, are expected to have a fairly homogeneous prescribing style. In this paper, we describe variation in prescribing style within and across groups of GPs from six countries.

**Methods:**

Cross-sectional study with the inclusion of 457 GPs and 6394 sore throat patients. We describe variation in prescribing antibiotics for sore throat patients across six countries and assess whether variation in “prescribing style” – understood as a subjective tendency to prescribe – has an important effect on variation in prescription of antibiotics by using the concept of prescribing style as a latent variable in a multivariable model. We report variation as a Median Odds Ratio (MOR) which is the transformation of the random effect variance onto an odds ratio; Thus, MOR = 1 means similar odds or strict homogeneity between GPs’ prescribing style, while a MOR higher than 1 denotes heterogeneity in prescribing style.

**Results:**

In all countries some GPs always prescribed antibiotics to all their patients, while other GPs never did. After adjusting for patient and GP characteristics, prescribing style in the group of GPs from Russia was about three times more heterogeneous than the prescribing style in the group of GPs from Denmark – Median Odds Ratio (6.8, 95% CI 3.1;8.8) and (2.6, 95% CI 2.2;4.4) respectively.

**Conclusion:**

Prescribing style is an important source of variation in prescription of antibiotics within and across countries, even after adjusting for patient and GP characteristics. Interventions aimed at influencing the prescribing style of GPs must encompass context-specific actions at the policy-making level alongside GP-targeted interventions to enable GPs to react more objectively to the external demands that are in place when making the decision of prescribing antibiotics or not.

## Background

Variation in prescription of antibiotics within and across countries is a problem of increasing concern [1-3] that needs to be seriously addressed in primary care as more than 80% of the antibiotics are prescribed at this level [4,5].

This variation can indicate poor clinical practice that increases the risk of adverse events for the patient [6], wastes health care resources [7] and contributes to the increase of resistant strains at the societal level [8].

The multidimensional causality of variation in prescription of antibiotics has been extensively studied during the last decade and two main approaches have been taken to find solutions. The first one is a population level approach, in which the determinants and in consequence the solutions are beyond the control of medical practice. It requires structural and cultural changes at a societal level [1,9,10].

The second one is an individual approach in which variation is caused by the characteristics of the patients [2], the General Practitioners (GPs) [11-13] and the organization of primary care services [14,15], and in consequence part of the solution is within the control of medical practice.

As part of this individual approach, previous qualitative and quantitative studies have shown that part of the variation can be explained by the subjective tendency that makes a GP to be more or less inclined to prescribe antibiotics [3,16-18]. Throughout the paper, we call this subjective tendency “prescribing style”.

Using Bourdieu’s theory of practice [19] for conceptualizing prescribing style, it can be defined as a *personal habitus* shaped in response to external demands, which results in a pattern of thinking and doing, without necessarily rational or conscious reasoning. Consequently, prescribing style is affected by the structural and cultural environment in which GPs work.

GPs as a professional group are expected to react very homogeneously to these external demands; hence, prescribing style reflects the extent to which GPs as a professional group adhere to objective criteria and have similar behavioural/psychological attitudes when making the decision to prescribe antibiotics or not.

In this paper, we assess whether variation in prescribing style is important to understand variation in prescription of antibiotics within and across groups of GPs from six countries when making the decision of prescribing antibiotics in patients with a sore throat. Prescribing style is operationalized as a latent variable(i.e. a variable that cannot be directly observed, although it can be inferred by the prevalence of prescriptions per GP and the residual variance), as proposed by Larsen et al. [20].

To the best of our knowledge, this is the first time that the magnitude of the variation in prescribing style is explored as a latent variable. It represents a new perspective in comparison to previous studies as it assesses the extent of homogeneity in the doing and thinking (practice) of GPs as a group, while taking into consideration patient and GP characteristics that have been previously associated with variation in prescription of antibiotics [2,12,15].

## Methods

### Design and setting

Cross-sectional study carried out in primary care in Argentina, Denmark, Lithuania, Russia (Kaliningrad), Spain and Sweden.

### Population

GPs and patients were part of the HAPPY AUDIT study (Health Alliance for Prudent Prescribing, Yield and Use of Antimicrobial Drugs in the Treatment of Respiratory tract Infections). HAPPY AUDIT was an EU-funded project aimed at promoting proper use of antibiotics across six countries by developing a quality circle between 2008 and 2009 [21]. This new analysis is based on data from the first data collection in 2008 and only includes GPs with 5 or more patients with a sore throat (pharyngitis and tonsillitis). The analysis is restricted to patients with a Sore throat as the modified Centor criteria (absence of cough, swollen and tender anteriorcervical nodes, temperature > 38°C, tonsillar exudates, age) [22] can be used to assess the adherence to objective criteria when making the decision of prescribing antibiotics or not. The patients represent 18.5% of all patients included in the HAPPY AUDIT study.

### Data collection

Data were collected during three consecutive weeks in the winter season of 2008. Two data collection instruments were used: a) a questionnaire about the organisation of primary medical care services completed by each GP, b) a chart registered by each GP each time they had a first encounter with a patient suspected of having a respiratory tract infection. The data collection instruments have been described in a previous article [21].

### Variables

The dependent variable in the multilevel model was the binary outcome “prescription of antibiotics” (yes/no).

To quantify “prescribing style”, we constructed hierarchical mixed-effect logit models with two levels: GPs and patients. For the GP level, we have two types of variables: I) Independent variables that in several models are included as fixed effects: a) GPs’ demographics (gender and age); b) professional experience (years as a practitioner); c) access to strep-A test as a tool to assess the presence of group A β-hemolytic streptococcus [22], d) organization of care (GP working in a solo or group practice). II) A random effect “prescribing style” that indicates the inherent tendency for each GP to prescribe antibiotics. The variance in these individual GP effects, beyond the influence of fixed effects in the model, measures individuality of the GPs: low variance indicates that GPs prescribe antibiotics similarly and tentatively adhere closely to objective criteria; high variance indicates that prescribing differs and the GPs do not adhere to objective criteria but more to their own behavioral/psychological attitudes. Hence, the variance of the random effect captures prescribing style of the GPs in the data.

Independent variables at patient level included: gender and age, patient expectations such as request of antibiotics, number of days with symptoms, and clinical characteristics (modified Centor criteria) used to evaluate the probability of bacterial origin of a sore throat [22].

### Statistical analysis

Baseline characteristics of GPs and patients were described as proportions and the percentage of patients receiving antibiotic prescriptions as medians (interquartile ranges). We developed three models to estimate variance in prescribing style (model A), and to investigate whether this variation is affected by patient characteristics (model B) and GP characteristics (model C).

The variance of the “prescribing style” random effect denotes variation between the GPs on a logit scale, but it is hard to interpret and cannot be directly compared to the magnitude of (fixed) effects of other variables. Therefore, we calculated a Median Odds Ratio (MOR), as proposed by Larsen et al. [20], which is a transformation of the random effect variance onto an odds ratio (OR) scale so that the magnitude of the variation can be compared to other effects that are expressed on an OR scale.

MOR can be interpreted by considering the selection of two GPs from the data and comparing their odds of prescribing antibiotics to a given patient. For such pair an OR is calculated, putting the GP with the higher prescribing tendency in the numerator (OR > 1). The median over all possible pairs of GPs is the MOR. Hence, MOR = 1 denotes equal odds or strict homogeneity between GPs in prescribing style. In contrast, a MOR > 1 means that GPs’ prescribing styles differ and are relevant for understanding variation in prescription of antibiotics.

**Model A** only includes the random “prescribing style” effect. As shown in previous studies [2,15,17], variation in prescription of antibiotics may be explained by patient and GP characteristics. Thus, we included in subsequent models a number of variables that could be confounders. **Model B** includes patient characteristics as covariates. Finally, **model C** includes both patient and GP characteristics.

A 95% confidence interval (CI) was computed for the MOR in each model with a parametric bootstrap. Resampled data sets were constructed by random sampling from the probability of prescription of antibiotics predicted by the corresponding model; the CI is calculated as the 2.5% and 97.5% percentile of the empirical distribution of the MORs calculated on 1000 of such resampled data sets. A similarly constructed CI for the difference between MORs from different models provides a test whether these MORs are different. This inference is made robust by omitting GPs with very few patients (<5).

The three models defined above and the corresponding MORs were estimated for each of the six countries separately. Descriptive analysis and multilevel modeling were performed with SAS version 9.3.

### Ethics statement

Data material is anonymous. The Happy Audit project (HA) was approved by The Scientific Ethical Committees from each country (Argentina: Medical association of General practice and family medicine, Misiones, Argentina. Denmark: The scientific ethical committee for Vejle and Funen counties, Odense, Denmark. Lithuania: Bioethics Committee of Klaipeda University, Klaipedia, Lithuania. Russia: Ministry of Health of the Government of Kaliningrad, Kaliningrad, Russia. Spain: Institut d'Investigacio Jordi Gol i Gurina, Barcelona, Spain. Sweden: According to Swedish legislation, ethical approval from the regional ethical review board was not needed for this study since it was part of a quality improvement activity).

Patients were informed about the objective of the project and were told that specific clinical information related to the consultation would be entered into a multinational database. Patients did not undergo any intervention, thus they were not asked to sign an informed consent.

## Results

Table 1 shows the baseline characteristics of the study population. A total of 6394 patients with sore throat were recruited by 457 GPs, 24.4% of the patients were under 15 year old. Only in Denmark and Sweden, 100% of the GPs had access to strep A test. Request of antibiotics varied across countries from 0.1% of the patients in Denmark to 9% of the patients in Russia.

**Table 1:** **Baseline characteristics of study populations**

	**ARG**	**DK**	**LT**	**RUS**	**SP**	**SW**	**Total**
**GPs/Patients (n)**	52/1054	64/614	28/584	30/550	257/3359	26/233	457/6394
***GP characteristics***							
**Female**	36(69)	31(48)	24(85)	26(86)	164(64)	9(34)	290(63)
**Age** = < 48y	41(79)	19(30)	13(46)	10(33)	128(50)	6(23)	217(47)
**No access to strep-A test** ^*^	41(80)	0	22(78)	28(93)	201(78)	0	292(63)
**Group practice**	25(48)	39(60)	26(92)	11(36)	232(90)	26(100)	359(78)
**Years working as a GP =** < 10	32(61)	28(43)	20(71)	21(70)	63(24)	11(42)	175(38)
***Patient characteristics***							
**Female**	580(55)	340(55)	270(46)	332(60)	2016(60)	116(49)	3654(57)
**Age** ^**†**^							
= < 2 years	111(10)	34(5)	58(10)	11(2)	36(1)	14(6)	264(4)
= > 3 to = <14 years	411(39)	197(32)	279(48)	155(28)	146(4)	109(47)	1297(20)
= > 15 to = <44 years	421(40)	311(51)	200(34)	293(53)	2071(61)	90(39)	3386(53)
>45 years	111(10)	72(12)	45(8)	91(16)	1099(33)	20(8)	1438(22)
**= < 3days with symptoms**	875(83)	388(63)	437(75)	427(78)	2464(73)	155(66)	4746(74)
**Request for antibiotics (yes)**	78(7)	1(0,1)	13(2)	49(9)	61(2)	10(4)	212(3)
**= > 2 Centor criteria** ^**‡**^	616(58)	317(51)	259(44)	293(53)	1242(37)	182(78)	2909(45)
**Patients prescribed antibiotics**	615(58)	285(46)	378(65)	377(68)	1386(41)	175(75)	3216(50)

Argentina (ARG), Denmark (DK), Lithuania (LT), Russia (RUS), Spain (SP), Sweden (SW).

n (%).

^*^Strep-A test: point of care diagnostic test employed to detect Group A β-hemolytic streptococcus.

^†^These age groups have a different risk for developing a bacterial sore throat.

^‡^Centor criteria: Fever > 38°C, absence of cough, tender anterior cervical adenopathy, tonsillar exudates.

Table 2 shows the ORs for prescribing antibiotics controlled by patient and GP characteristics. In general, characteristics at GP level as well as demographic characteristics of the patients were not associated with prescription of antibiotics.

**Table 2:** **Multilevel logistic regression for the association of patient and GP characteristics with prescription of antibiotics**

	**ARG**	**DK**	**LT**	**RUS**	**SP**	**SW**
**GPs/Patients (n)**	52/1054	64/614	28/584	30/550	257/3359	26/233
**GP level**						
Male vs Female	3 (0,9;10,4)	1,6 (0,7;3,5)	0,3 (0,03;3,8)	0,5 (0,03;8,7)	1 (0,6;1,6)	1 (0,1;6,2)
Age (= > 49y vs = < 48y)	1,2(0,2; 6,7)	0,8(0,3;2,5)	1,5(0,3;7,1)	8,3(0,7;91)	1(0,6;1,6)	2,1(0,1;29)
Access to strep A test (Yes vs No)	1,3 (0,3;5,3)	N/A	1,8 (0,3;10,7)	12 (0,1;1137)	1,7 (1;2,8)	N/A
Years working as a GP (= > 11y vs = < 10y)	1,3(0,2;6)	1,2 (0,4;3)	0,05 (0,01;0,3)	0,2 (0;42)	1,3 (0,7;2,3)	0,4 (0,04;3,6)
Type of practice (solo vs group)	0,8 (0,2;2,7)	0,8 (0,4;1,7)	1,3 (0,08;22)	N/A	0,5 (0,2;1)	N/A
**Patient level**						
Male vs Female	0,6 (0,4;1)	0,9 (0,6;1,4)	0,8 (0,5;1,3)	0,9 (0,4;2)	0.9 (0,7;1,1)	1,1 (0,4;3,1)
Age = < 2 years vs						
= > 3 to = < 14 years	1,7 (0,8;3,3)	0,9 (0,3;2,3)	1,8(0,8;3,8)	0,5(0,06;4,4)	0,8 (0,2;2,6)	0,3 (0,03;3,1)
= > 15 to = < 44 years	2,7 (1,3;5,6)	0,8 (0,3;2)	1,5 (0,6;3,5)	0,7 (0,08;6,8)	0,8 (0,2;2,9)	0,5 (0,06;5,1)
= > 45 years	2,1 (0,8;5,4)	0,8 (0,3;2,4)	1,3 (0,4;4,3)	0,9 (0,09;9)	0,6 (0,1;2,1)	0,3 (0,03;4,8)
Number of days with symptoms (= < 3d vs= > 4d)	1,2 (0,7;2,2)	0,8 (0,5;1,2)	1,5(0,8;2,7)	6,5(2;20)	1,1(0,9;1,5)	0,7(0,2;2)
Request for antibiotics (No vs Yes)	15,6 (5;48)	N/A	N/A	8 (2;33)	9,7 (4,5;21)	N/A
Number of Centor criteria(<2vs= > 2)^‡^	16,5 (10;25)	6,7 (4,2;10)	13,8 (7;27)	42 (17;104)	34 (25;44)	21 (6,5;70)

Argentina (ARG), Denmark (DK), Lithuania (LT), Russia (RUS), Spain (SP), Sweden (SW).

^‡^ < 2 = 0 or 1 Centor criteria.

Mutually adjusted odds ratios.

N/A = variable did not fit in the model.

Only in Russia, 4 or more days with symptoms was positively associated with prescription of antibiotics (odds ratio 6.5, 95% confidence interval 2 to 20). Furthermore, in Argentina, Russia and Spain, patient request for antibiotics was positively associated with prescription of antibiotics.

**Figure 1:**
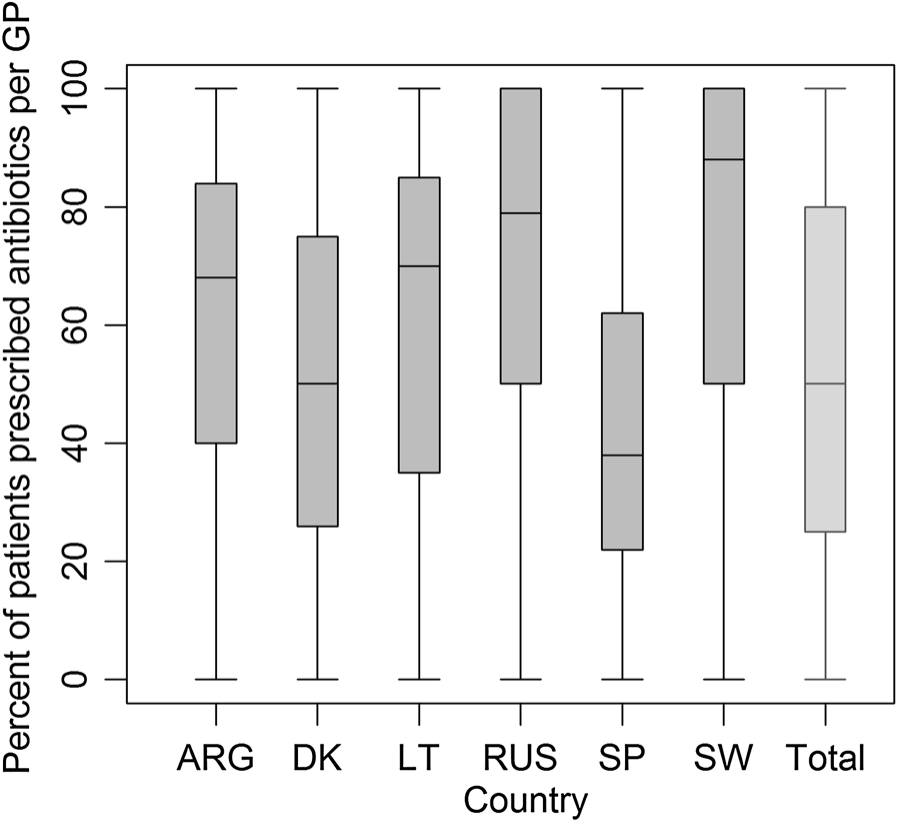
**Crude variation in prescription of antibiotics per country.** Box-and-whisker plot shows proportions of patients prescribed antibiotics per country. The horizontal line inside the box shows the median percentage of patients prescribed antibiotics for sore throat and the upper and lower end of each box give the 75^th^ and 25^th^ interquartile ranges, respectively. The area between the different parts of the box indicates the degree of dispersion and skewness of data. The ends of the whiskers represent the maximum and minimum percentage of patients that were prescribed antibiotics.

Figure 1 shows the crude variation in the prescription of antibiotics within and across countries. The median percentage of patients being prescribed antibiotics varied across countries from 38% (interquartile range (IQR) 22%-62%) in Spain to 88% (IQR 50%100%) in Sweden. There was variation in prescription of antibiotics within the countries too, represented by the asymmetry of the interquartile ranges. In all countries some GPs always prescribed antibiotics to all their patients while other GPs never did.

**Figure 2:**
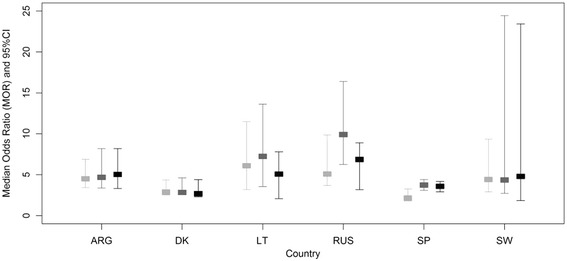
**Unadjusted and adjusted Median Odds Ratios (MOR) per country.** The diagram shows the multilevel analysis of the variance of GPs’ prescribing style. Model A (light grey): prescription of antibiotics is only a function of GPs’ prescribing style. Model B (medium grey): prescription of antibiotics is a function of GPs’ prescribing style and patient characteristics. Model C (dark grey): prescription of antibiotics is a function of GPs’ prescribing style, patient and GP characteristics. When MOR = 1, there is no variation in GPs’ prescribing styles. The higher the MOR, the more variation in GPs’ prescribing styles.

Figure 2 shows the multilevel analysis of the variance of GPs’ prescribing style (model A) and the changes after adjusting for patient characteristics (Model B) and afterwards adding GP’s characteristics (Model C).

After adjusting by patient and GPs’ characteristics (model C), the Median Odds Ratio was consistently greater than 1 within countries and varied across countries. The most heterogeneous group of practitioners was found in Russia (Kaliningrad) Median Odds Ratio (MOR 6.8, 95% CI 3.1; 8.8). It means, in Russia a randomly chosen patient has a median 6-fold risk of being prescribed antibiotics if consulting a GP with a higher tendency to prescribe antibiotics.

The group of Danish GPs had the most homogeneous prescribing style (MOR 2.6, 95% CI 2.2; 4.4).

## Discussion

### Summary of main findings

In this paper we described the variation in prescription of antibiotics for patients with a sore throat within and across groups of GPs from six countries. We used the concept of the latent variable to assess variation in prescribing style (model A), and to investigate whether this variation was affected by patient characteristics (model B) and GP characteristics (model C).Variation was ubiquitous within and across countries. In all countries some GPs always prescribed antibiotics to all their patients, while other GPs never did.

After adjusting for patient and GP characteristics, variation in GPs’ prescribing style was consistently large – between MOR = 2 and MOR = 6 –. It indicates heterogeneity within and across countries in the pattern of thinking and doing when making the decision of prescribing antibiotics or not to patients with a sore throat.

### Interpretation

As reported in previous studies, we found variation in antibiotic prescriptions within countries [3,17] and across geographical regions [2,23]. After adjusting for patient and GP characteristics, variation in GPs’ prescribing style was consistently large, which in line with other studies [3,17] confirms that prescribing style is a personal tendency that influences the variation in prescription of antibiotics.

To the best of our knowledge, this is the first time that prescribing style is measured within and across countries in a way that allows assessment of the heterogeneity of GPs as a group.

GPs from Russia, Lithuania and Argentina had the most heterogeneous prescribing style. These groups of GPs struggle with common external factors such as weak political leadership to encourage antibiotic stewardship and weak surveillance of the over-the-counter sale of antibiotics [24,25]. It could indicate that the personal tendency that makes GPs to have a very heterogeneous prescribing style is highly influenced by policy-making factors at a societal level.

Furthermore, GPs from these countries were exposed to a higher percentage of patients requesting antibiotics, most of them had not access to strep-A test and they did not have national guidelines for the management of sore throat patients. It could indicate that the large variation in the groups of GPs from these countries is not only related to policy-making factors at a societal level, but also to the lack of adherence to common objective criteria. Also different behavioural/psychological attitudes may affect ability to cope with the uncertainty of the bacterial origin of symptoms and the pressure from the patients.

We found variation within and between the group of GPs from Denmark and Sweden. The GPs from these two countries have in common that they work in an environment with a strong political leadership regarding antibiotic stewardship [5,26] and have guidelines for the management of sore throat patients.

There are two important factors that can explain this heterogeneity. Firstly, as shown in previous studies [13,16,27], it indicates that personal psychological/behavioural attitudes towards uncertainty and risk, at GP-level, are important to understand variation within GPs.

Secondly, the level of adherence to common objective criteria depends on knowledge exchange and appropriation of the knowledge as a group. In a recent qualitative study that explored variation in the management of patients with a sore throat [16] in a group of Swedish GPs, they found that GPs could be divided into two groups: those who fully adhere to the guidelines and other group who did not follow the guidelines in spite of knowing them. The main difference between these two groups was that those in the adherent group used to meet and discuss about the guidelines while those in the non-adherent group did not use to discuss their knowledge with their colleagues.

Sharing knowledge by open discussion between peers has been one of the core strategies for quality development that have been promoted for the Audit Project Odense (APO) during the last 25 years in Denmark, thus most of the Danish GPs that took part in the HAPPY AUDIT were used to participate in knowledge-exchange networks.

### Limitations

The generalizability of the findings has to be interpreted with caution. The APO methodology relies on voluntary participation and there is evidence that the prescription rate of GPs that participate in Audits differs from the prescription rate of GPs that do not participate in such activities [28]. Thus, the sample of GPs may not be representative of the countries’ GP population. In any case, it could mean that the estimated variation within and across countries is quite conservative in comparison to the variation between the whole population of GPs from each country.

There could be ascertainment bias. Although a common data collection instrument was developed by representatives from each country and carefully translated into each language and back to English to double-check and minimise the risk of misunderstandings, we cannot rule out differences in the interpretation of the diagnostic criteria for sore throat due to language and cultural context differences [29].

### Perspectives

Promotion of proper use of antibiotics, as a key strategy to curb the spread of antibiotic resistance strain, needs of innovative starting points that can bring together the population and individual level sources of variation in prescription of antibiotics within and across countries.

This new perspective that gives the GPs the opportunity to assess their heterogeneity as a group could empower them to advocate for structural changes at the societal level and look for solution as a group to decrease heterogeneity, while decreasing the misuse of antibiotics.

The use of the MOR as a measure of group variation needs of further validation in larger groups of GPs and ideally using data from databases to assess the real prescription pattern of GPs not influenced by the participation in Audit activities.

Finally, future research could focus on the extent to which success of interventions aimed at promoting proper use of antibiotics could be assessed by measuring the decrease in the variation of GPs as a group.

## Conclusion

GPs as members of a professional group react heterogeneously to the external demands that are in place when making the decision to prescribe antibiotics or not; thus playing a key role in the variation in the prescription of antibiotics within and across countries.

Interventions aimed at promoting proper use of antibiotics should encompass actions at the policy-making level alongside GP-targeted actions that focus on knowledge appropriation; as well as capacity to deal with uncertainty, to enable GPs to react more objectively when making the decision of prescribing antibiotics or not.

## Abbreviations

GPs: General practitioners
MOR: Median Odds Ratio
HAPPY AUDIT: Health Alliance for Prudent Prescribing, Yield and Use of Antimicrobial Drugs in the Treatment of Respiratory tract Infections
